# 

**Published:** 1874-01

**Authors:** 


### CONTENTS:

### ORIGINAL COMMUNICATIONS.

ARTICLE I.—The Larynx the Source of the Vowel Sounds. By Thomas Brian Gunning, D. D. S., M. D.......

### SELECTED ARTICLES.

ARTICLE II.—Death from Ether in a Dental Office.......

ARTICLE III.—Oral Surgery.......

ARTICLE IV.—Vulcanite Litigation.......

### EDITORIAL, ETC.

Blindness Cured by the Extraction of Diseased Teeth.......
Metallic Enamel.......
The Dental Rubber Cases—A Novel Law Suit.......
Webster's Unabridged Dictionary.......

### MONTHLY SUMMARY.

Listening with the Teeth.......
The Prevention of the Loss of Blood.......
Mercury in the System.......
Lead Poisoning through Drinking Water.......
Hope for the Bald.......
Modification of the Operation for Harelip.......
Bad Tea.......
Facial Paralysis.......
Surgical Use of Gastric Juice in Treatment of Cancerous Tumors.......
Test for Impurities in Water.......

